# Doping of Magnéli Phase—New Direction in Pollutant Degradation and Electrochemistry

**DOI:** 10.3390/molecules30214282

**Published:** 2025-11-04

**Authors:** Vanja Vojnović, Maja Ranković, Anka Jevremović, Nataša R. Mijailović, Bojana Nedić Vasiljević, Maja Milojević-Rakić, Danica Bajuk-Bogdanović, Nemanja Gavrilov

**Affiliations:** 1 Institute of Chemistry, Technology and Metallurgy, University of Belgrade, Njegoševa 12, 11000 Belgrade, Serbia; vanja.vojnovic@ihtm.bg.ac.rs; 2 University of Belgrade-Faculty of Physical Chemistry, Studentski Trg 12-16, 11158 Belgrade, Serbia; maja.rankovic@ffh.bg.ac.rs (M.R.); anka@ffh.bg.ac.rs (A.J.); bojana@ffh.bg.ac.rs (B.N.V.); maja@ffh.bg.ac.rs (M.M.-R.); danabb@ffh.bg.ac.rs (D.B.-B.); 3 Department of Pharmacy, Faculty of Medical Sciences, University of Kragujevac, 34000 Kragujevac, Serbia; nacakg@gmail.com

**Keywords:** titanium suboxide, Magnéli phase, pollutant, degradation, electrochemical oxidation

## Abstract

This review summarizes the recent developments in titanium suboxide (TSO) doping and the application of doped materials in pollutant degradation and electrochemistry. Doping is mainly limited to transition and rare-earth metals, with some exceptions, of similar ionic radii and charge, that can replace Ti ions in TSO without too much disturbance to the lattice. Consequently, doping is limited to below 10 at%, which predominantly induces oxygen vacancy formation. Doping mechanisms are weighted, and their effect on conductivity, stability, and catalytic activity is overviewed. High-temperature H_2_ reduction of TiO_2_ is still the dominant preparation method, with carbothermal reduction and Ti reduction gaining ground due to safety and energy concerns. Doping predominantly increases the conductivity 2–5 times, while the stability can be both improved or worsened, depending on the size and charge of the doping ion. Electrochemical oxidation, at positive overpotentials, of per- and polyfluoroalkyl substances (PFAS), antibiotics, and other water pollutants, is the main avenue of application. Doping almost exclusively leads to complete selected pollutant degradation and improvement of the pristine TSO, which is summarized in detail. New niche applications of peroxide, hydrogen, and chlorine production are also viable on doped TSO and are touched upon. Complementing experimental results are theoretical calculations, and we give an overview of density functional theory (DFT) results of transition metal-doped TSOs, identifying active centers, degradation trends, and potential new doping candidates.

## 1. Introduction

Titanium oxides represent a versatile class of inorganic materials with a wide range of applications. Among them, titanium dioxide (TiO_2_) is the most thermodynamically stable phase with distinguished chemical stability, biocompatibility, abundance, and low cost [1,2,3]. TiO_2_ finds applications in diverse fields, such as reducing the toxicity of dyes and pharmaceutical drugs [4], wastewater treatment [5,6], sensing [7], agriculture [8], air purification [9], and solar cells [10]. Owing to its semiconducting nature, TiO_2_ is extensively utilized in photocatalysis [11,12].

Under reductive conditions or controlled thermal treatment in reducing atmospheres, titanium dioxide can be transformed into a series of oxygen-deficient, non-stoichiometric titanium suboxides known as Magnéli phases (MPs), with the general chemical formula Ti_n_O_2n−1_, where n typically ranges from 3 to 10 [13,14,15,16]. These suboxides, often commercially recognized under the trade name Ebonex^®^ [17], have gained researchers’ attention for their unique physicochemical properties [18,19].

While stoichiometric TiO_2_ is a wide-bandgap semiconductor with extremely low electrical conductivity in ambient conditions (10^−13^ to 10^−14^ S/cm), Magnéli phases exhibit semi-metallic or metallic-like behavior [15,20]. These oxygen-deficient titanium suboxides (TSOs) possess significantly enhanced electrical conductivity, often comparable to that of carbon, and display excellent chemical and thermal stability, making them highly attractive for a range of energy and environmental applications. The value of n strongly influences the electrical conductivity of Magnéli phases, reaching its maximum for n = 3–5, while higher values are associated with a progressive reduction in conductivity [15,21]. Among these, Ti_4_O_7_ stands out due to its exceptionally high electrical conductivity (reaching approximately 1.6 × 10^3^ S/cm at ambient temperature), comparable to graphite (∼1000 S/cm) [13]. Consequently, Magnéli phases, especially Ti_4_O_7_, are being increasingly investigated as electrode materials for electrochemical applications due to their superior conductivity and enhanced thermal and chemical stability [15].

Reduced titanium oxides have been investigated for use in various applications, including electrode materials [18,22,23], thermoelectric devices [24,25], and photocatalysts [16].

The crystal structure of the Ti–O Magnéli phase is primarily composed of Ti–O octahedra similar to those in rutile TiO_2_, featuring regularly arranged planar oxygen vacancies occurring every n layers of Ti–O octahedra as a result of missing oxygen atoms [26].

**Figure 1:**
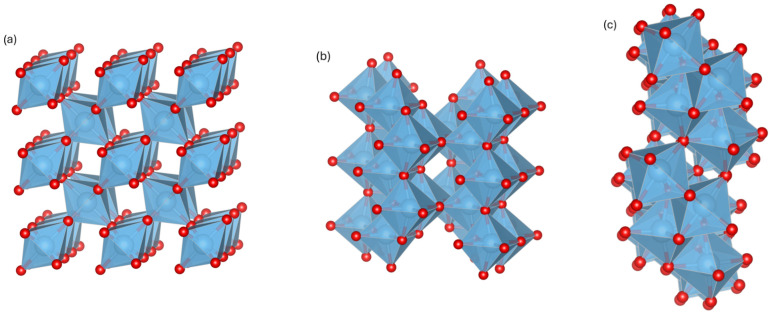
Crystal structure of original rutile (**a**) TiO_2_, (**b**) Magnéli Ti_3_O_5_, and (**c**) Magnéli Ti_4_O_7_. Red: oxygen atoms; blue: titanium atoms.

Magnéli and co-workers used XRD to study transition-metal oxides (Ti, V, Mo, W) and confirmed the homologous series Ti_n_O_2n−1_ (n = 2, 3, …), all derived from the rutile structure. Wadsley later introduced the concept of crystallographic shear (CS) structures, formed by block division, shear translation, and removal of overlaps. For Ti_n_O_2n−1_ (n = 2–10), the CS plane and vector were identified as (121) and $½[20\bar{1}]$ in rutile structure, while for n > 10, Bursill et al. [27] reported a shift to the (132) rutile plane [24]. As the n value in MPs decreases, the interspacing between the CS plane contracts, leading to an increase in oxygen-vacancy density and an increase in the number of Ti^3+^ centers. The vacancy-derived Ti_(d)_ defect states are located close to the conduction-band minimum and serve as intrinsic dopants, facilitating electron delocalization across the CS plane structure and thus improving the conductivity of MPs with lower n [28]. Half of the oxygen vacancies corresponds to the concentration of delocalized carriers with vacancy density (and n) directly affecting electrical conductivity [29]. Ti_4_O_7_ is the first member and possesses the highest electrical conductivity among the MP family of titanium suboxides. For example, Ti_3_O_5_ does not classify as an MP because of the lack of ordered shear planes in its crystal structure, preventing it from obtaining the same extensive vacancy-band conduction. Experiments and DFT+U studies on reduced Ti-suboxides demonstrate that the formation of Magnéli phases with an intermediate average Ti valence (^3+^/^4+^), particularly Ti_4_O_7_ with equal Ti^3+^/Ti^4+^ (Ti^3.5+^), results in fully delocalized d-electrons and metallic-like transport [20]. The crystal structure of Ti_4_O_7_ belongs to the triclinic P-1 space group. It can be described as rutile blocks four octahedra wide, separated by crystallographic shear planes. These blocks are shifted relative to one another, creating chains of edge-sharing TiO_6_ octahedra (Figure 1) [30].

Magnéli phase TSOs can be synthesized either by heating TiO_2_ with metallic titanium in an inert atmosphere according to the following reaction:

(1) $$(1/2)\mathrm{Ti}\;+\;(n\;-\;(1/2)){\mathrm{TiO}}_{2}\;\to {\mathrm{Ti}}_{n}O_{2n-1}$$

or by reducing TiO_2_ at elevated temperatures using a reducing agent such as hydrogen gas, following the reaction

nTiO_2_ + H_2_ → Ti_n_O_2n−1_ + H_2_O (2)

Upon reduction, rutile TiO_2_ undergoes a stepwise transformation through a series of MP TSOs, progressing as follows: TiO_2_ (tetragonal) → Ti_9_O_17_ (triclinic) → Ti_8_O_15_ (triclinic) → Ti_6_O_11_ (triclinic) → Ti_5_O_9_ (triclinic) → Ti_4_O_7_ (triclinic) → Ti_3_O_5_ (monoclinic) → Ti_2_O_3_ (trigonal) [31].

TiO_2_ is commonly used as the primary precursor due to its availability and low cost. The reduction typically requires temperatures above 1273 K and a reducing atmosphere, usually hydrogen [13,15]. The heat treatment induces defects in TiO_2_’s lattice, including oxygen vacancies, which alter the titanium-to-oxygen ratio, resulting in the formation of different suboxide phases [32]. Ti_4_O_7_ can be synthesized using a molten salt method, yielding rod-like particles as suggested by [26]. A bulk MP prepared by spark plasma sintering (SPS) shows high conductivity (over 950 S/cm), significantly higher than MPs from conventional routes. Another SPS route combined with sol-gel synthesis is applied for the preparation of MP nanocomposites (Ti_n_O_2n−1_), both as pristine nano-MPs (n = 3, 4, 5, 6, 8) with up to 300 m^2^/g, low resistivity, and thermal conductivity [33]. Zhang et al. [34] densified Ti_4_O_7_ powders with particle sizes of 1–2 μm nearly to full density at 900 °C under a 40 MPa axial pressure using SPS. However, even under vacuum during SPS, the Ti_4_O_7_ completely oxidized to Ti_5_O_9_. Yu et al. report the densification of Magnéli phase titanium suboxide powders using Flash SPS, aiming to preserve the original Ti_4_O_7_ phase and achieve a uniform microstructure in the sintered samples [35]. Hydrothermal reactor surface areas have a substantial influence on hydrothermal synthesis. Low-temperature (only 363 K) triclinic MP Ti_6_O_11_ was obtained with hierarchical morphology [36]. Additionally, Magnéli phases were obtained via hot-pressed sintering of single-phase submicron Ti_4_O_7_ powders [37], hot-pressed sintering TiO_2_ and Ti powder under a vacuum [38], and through high-pressure torsion [39].

MP materials are highly valued for their exceptional chemical inertness, durability, and resistance to corrosion in aggressive environments. For example, Ebonex^®^ exhibits remarkable stability, with a projected half-life of 50 years in 4 M sulfuric acid at ambient temperature [15,40]. The rutile-type surface of MP ceramics serves as an advanced support for various catalytic coatings [15], while the addition of transition metals can lead to improved catalytic performance.

Doping is a powerful strategy for tailoring the properties of Ti_n_O_2n−1_ phases for specific applications. The incorporation of different dopants has been shown to alter the electrical and thermal properties, thereby enhancing photocatalytic, thermoelectric, and electrocatalytic performance. However, a dopant selection is often limited to size and favors high solubility of solutes in metal alloys. Dopants with high solubility reduce the risk of forming secondary phases, which are difficult to detect at low concentrations. Studies suggest that the low electronegativity difference between solute and host with similar coordination number and the atomic radii within ±15% in similar crystal structures should be followed, while relaxing the electronegativity constraint to not more than ±0.4 on the Pauling scale [41]. This refinement of potential dopant lists provides a basis for studying their influence on the transformations between Ti_n_O_2n−1_ and TiO_2_. However, although Pauling’s rules provide a useful framework for describing MP structures, their predictive capability may be limited. George et al. [42] reported that only a small fraction of metal oxides conform to these rules, indicating that more quantitative predictors of crystal stability should be taken into account.

Successful doping resulting in enhanced materials does not warrant better application performance; thus, doping methods are still under consideration by researchers, especially those oriented toward sustainable water treatment solutions. The emergence of Magnéli doping and its application prompted us to review this emerging niche briefly. Recently, several reviews on the Magnéli phase topic have been published [42,43,44,45]. However, this paper provides a review of the enhancements of doped Magnéli phases for environmental and energy applications.

## 2. Synthesis of Doped Magnéli Phases

The preparation of doped Magnéli phases requires controlled replacement of the dopant element into the lattice with concurrent oxygen vacancy formation. Traditionally, this is performed at high temperatures (1173–1473 K) using hydrogen, carbon, or titanium as reducing agents, as the most reliable approach for achieving homogeneous dopant distribution and stable phase formation. These conditions facilitate the diffusion of dopant ions into Ti sites and the generation of oxygen vacancies necessary for charge compensation. Doping is often achieved using a transition metal precursor, while recent studies investigated rare-earth dopants for MP structures (e.g., La, Ce, Nd, and Sm). These dopants influence the electronic structure and defect chemistry in MPs, as an alternative route for tuning their activity. Additionally, recent studies have demonstrated that solution-based methods, including solvothermal and hydrothermal reduction, can yield partially reduced TiO_2_ structures that evolve into doped Magnéli-like phases upon mild post-annealing. Such approaches enable better control of particle morphology, dopant valence states, and local defect concentration. However, achieving complete phase transformation and avoiding dopant segregation remain key challenges for low-temperature synthesis. A combined strategy that integrates low-temperature dopant incorporation with subsequent controlled annealing may therefore represent a promising route toward scalable, compositionally tunable Magnéli materials.

An elegant selection of dopants relies on their ionic radius, electronegativity, thermodynamic properties, and electronic environment. Metals with a coordination number of 6, a trivalent oxidation state, and a corundum-like crystal structure are good candidates for doping. Thus, Fe, V, and Cr, with ionic radius 60–75 pm (near the value for Ti (III/IV), 61–74 pm) and electronegativity 1.9–1.6, were the starting point for experimental studies. The substitution of Ti by other transition-metal dopants such as V or Ce is also governed by the balance between ionic radius compatibility and oxidation state stability. Vanadium, with a comparable ionic radius (V^4+^ = 72 pm) compared to Ti^4+^ (74 pm), can occupy Ti sites with minimal lattice distortion while promoting localized Ti^3+^ formation to preserve charge neutrality. In contrast, Fe^3+^ (60 pm) substitution induces more apparent lattice strain and facilitates the evolution of oxygen vacancies, acting as electron donors and enhancing electrical conductivity. Ce incorporation, due to its flexible Ce^3+^/Ce^4+^ redox couple and larger ionic radius (102 pm for Ce^3+^, 101 pm for Ce^4+^), introduces significant local distortion but contributes additional oxygen vacancy pathways through redox exchange. The dopant effects modify the Ti^3+^/Ti^4+^ ratio and the density of defect states near the Fermi level, which in turn influence charge transport and surface reactivity. Consequently, the electrochemical performance of doped Magnéli phases can be directly correlated to the interaction between dopant-induced lattice distortions, oxygen vacancy formation mechanisms, and changes in Ti valence distribution.

To resolve a mechanism for thermal oxidation and investigate the influence of doping, the thermal oxidation behavior in air of Ti_4_O_7_ doped with V, Cr, and Fe was investigated by English and Wilkinson [41] using thermogravimetry. Materials were prepared by high-temperature H_2_ reduction of dopant-containing TiO_2_. V- and Fe-doping improved the thermal stability of Ti_4_O_7_, while diffusion reaction models described the solid-state kinetics of oxidation. The estimated material lifetimes show a decrease with Cr doping, although the amount was kept at 2% despite its optimal radius and crystal structure. The MP electrodes were also synthesized by mechanically activating rutile with Ti and Nb, V, and Fe additives, followed by sintering in reducing or inert atmospheres up to 1353 K [21]. Mixed V/Ti V_n−x_Ti_x_O_2n−1_ Magnéli phases were synthesized under vacuum, starting from commercial V, V_2_O_5_, and Ti_2_O_3_, as powders and single crystals grown by chemical vapor transport [46]. Ti incorporation minimally affects the vanadium oxide structure but suppresses its metal–insulator transitions. The structure and properties of the obtained material, such as the presence of crystalline phases and thermoelectric characteristics, will depend on the doping level. A series of reduced Ti_1−x_Nb_x_O_2−_δ materials was synthesized by conventional solid-state reduction in a 10% H_2_ below 1480 K [47]. Compositions up to x = 0.01 contained mixed anorthic phases (Ti_n_O_2n−1_, 8 ≤ n ≤ 10) with additional Ti_10_O_18_ reflections, whereas the x = 0.02 sample showed rutile TiO_2_, and higher Nb concentrations (x = 0.04 and 0.08) stabilized the rutile phase exclusively. The optimum thermoelectric response was obtained at x = 0.01, with a ZT value of 0.023 at 380 K, which is almost 2.6 times higher compared to the undoped composition.

Several other elements have also been investigated as dopants. For example, Ga(III) and Mn(IV), but they exhibited drawbacks in terms of stability and feasibility [41,48].

Gardos tested Cu, Fe, Co, and Ni ions as dopants, introduced in the form of their stable oxides through a simple ball-milling, hot-pressing, and annealing process, but only the (Ti + Cu)O_1_._80_ model mixture yielded the desired reaction. Hot-pressing anatase/CuO powders with subsequent heating in a 3% H_2_ atmosphere resulted in a portion of Cu entering the rutile-transformed lattice to form a titanium–copper crystallographic shear-induced MP [49]. The alloying effect of ZrO_2_ on the microstructure of MP was also studied. Magnéli contains periodic shear planes in its rutile matrix, and ZrO_2_ has a rutile structure—6 mol% of dopant enables homogeneous Zr distribution [50].

Ti_4_O_7_ powders with varying Ce doping levels can be synthesized via a wet-chemical route combined with hydrogen reduction. Ce is incorporated into the lattice exclusively as Ce^3+^, with a solubility limit below 9 at%. Electrical conductivity decreases with Ce doping due to reduced electronic states near the Fermi level [51]. Sn-doped MP was prepared by polymerization–calcination and surface-loading Sn onto MP via grinding–calcination. Doping induced higher conductivity, lattice distortion, and increased oxygen vacancies/Ti^3+^, while surface doping favors the 4e^−^ pathway due to surface Sn–Ti interactions [52]. Table 1 summarizes the applications or the alterations in the structural and thermal characteristics of doped MP.

## 3. Electrocatalytic Applications

Due to their intrinsic conductivity and relative stability, doped Magnéli phases have found numerous applications in (electro)catalysis, particularly in pollutant degradation and other electrochemical fields.

Industrial wastewater often contains contaminants that necessitate pretreatment before being released into biological treatment systems and natural water bodies. Magnéli phase titanium oxides were doped with V, Fe, and Cr to assess their suitability for the electrochemical oxidation of industrial wastewater [56]. Conductivity increased slightly upon doping from 120 S/cm to between 300 and 380 S/cm. The V effect corresponds to one seen for Fe-doping, which outperforms Cr-dopingand pristine MP. Additionally, V and Fe improved the thermal stability of MP in air. However, Cr doping of MP decreased its oxidative stability, while V doping improved it and was therefore chosen for the testing of industrial wastewater degradation. V-doped MP achieved complete pollutant degradation and diminished chemical oxygen demand, which was attributed to its greater electrochemically active surface area and improved anodic stability, compared to pristine MP.

Pharmaceuticals often resist degradation, and electrochemical methods with suitable electrode materials are investigated in this area. Tao et al. [57] prepared Sm-doped Ti_4_O_7_ for anodic decomposition of sulfamethazine with three times higher degradation efficacy over pristine Ti_4_O_7_ and 91% overall degradation. Doping with Sm shifted the electro-generation of the hydroxyl radical to a more positive potential, increasing the electro-oxidation efficacy and effectiveness. Sn doping of Ti_4_O_7_ was prepared by Jia et al. [59] via high-temperature H_2_ reduction for wastewater antibiotic (tetracycline) degradation. At low dopant concentration (<1%), a tetracycline degradation of 92% was achieved after 2 h, an improvement upon 71% from the pristine Magnéli phase, and complete removal after 3 h. Kao [68] conducted La, Al, and Co doping of Ti_4_O_7_ for the degradation of pharmaceutics. Among the four studied drugs (cyclophosphamide, carbamazepine, sulfamethoxazole, and ibuprofen), cobalt-doping proved to be the most effective for almost complete sulfamethoxazole removal within 60 min. However, leaching of Co was evidenced, and the lifetime of the electrode was limited to 30 h of operation.

Jing et al. [58] successfully prepared Co-doped TSO via a simple ball milling procedure with no oxide impurities. The material was proven to completely remove sulfamethoxazole in wastewater at high effluent fluxes with a large rate constant. Strong Co-TSO interaction is cited as the reason for its stability, and Co proclivity towards forming OH-radical and ^1^O_2_ as the reason for the high rate constant.

Chen et al. [60] simultaneously doped MP with Ce and composited with carbon black (CB) in an effort to achieve monocycline degradation within wastewater. Complete removal was achieved within 20 min, but the electrode efficiency dropped to 98.5% after only five cycles. OH-radical and ^1^O_2_ were identified as the dominant species responsible for monocycline degradation, similar to [58].

Another persistent class of compounds is per- and polyfluoroalkyl substances (PFAS). Recently, Sui et al. [55] performed solid-state Ce and Nb Ti_4_O_7_ doping for the degradation of perfluorooctane sulfonate (PFOS) through electrochemical oxidation. Nb doping was put forward for PFOS degradation due to its lower charge transfer resistance and higher effective electrochemical surface area (EESA). Ce doping led to lower EESA and lower activity for PFOS degradation, but when normalized with respect to EESA, it also showed improvement compared to pristine Ti_4_O_7_. Listed recent studies point to doped-TSO being predominantly used for pollutant degradation: PFAS/PFOS, antibiotics, pesticides, microbe inactivation, and overall (waste)water purification. The main mechanism includes OH-radical and ^1^O_2_ generation at higher potentials, which can directly, or in an indirect way, degrade target molecules. Shifting the oxygen evolution potential to more positive values is the main goal, as it improves the degradation process efficacy.

Other niche applications, including bacteria inactivation and H_2_ and Cl_2_ production towards health and safety, are also being considered.

Zhang et al. [63] 3D printed Nd-, Sm-, and Pr-doped TSO and tested it for *E. coli* inactivation. Printed reactive electrochemical membranes increased hydroxyl radical yield by 50–450% compared to pure Ti_4_O_7_.

Wierzbicka et al. [53] prepared a mixed-phase TiO_2_-based catalyst with 32% anatase, 11% rutile, and 57% Magnéli phases loaded with Pt for H_2_ evolution. Higher H_2_ production, between 50–100 times that of plain anatase loaded with a similar amount of Pt, was reported, which was attributed to a multijunction of various Magnéli titania species and Pt.

The production of peroxide as a disinfectant, with H_2_ and Cl_2_ as desired base chemicals, is another avenue of doped-TSO application that is gaining attention due to doped-TSO’s concurrent activity and stability. Sun et al. [52] successfully incorporated Sn into the bulk of TSO and utilized it for the selective reduction of O_2_ to peroxide. The onset potential for peroxide evolution was close to theoretical in 0.1 M KOH, thanks to the abundance of oxygen defects resulting from Sn incorporation with a selectivity of 96 %. Lee et al. [62] incorporated Ru inside TSO, forming surface Ru-O_4_ motifs via a wet impregnation and mild annealing for the chlorine evolution reaction. Low-level Ru doping (0.13 wt%) provided outstanding activity and selectivity toward the chlorine evolution reaction due to direct and selective Cl^−^ adsorption on Ru-O_4_ motifs.

## 4. Theoretical Insights

Doped MPs have been treated by density functional theory (DFT), with results indicating that doping with transition metals or non-metals can further tune their electronic structure, conductivity, and catalytic activity. Detailed analysis of how dopant incorporation modifies the local environment around crystallographic shear planes, alters the distribution of oxygen vacancies, and influences the density of states near the Fermi level is still lacking. Such insights would be of great significance for optimizing doped MPs.

The work by Yuan et al. provides guidelines for designing high-performance doped Ti_4_O_7_. Namely, by using DFT and machine learning, the stability and electronic structure of doped (Ti, M)_4_O_7_ (M = Sc, Y, La, Ce, Zr, Hf, V, Nb, Ta, Cr, Mo, W) were examined. Although doped MP are thermodynamically stable, Y, La, and Ce introduce significant lattice distortion, while Zr, Nb, Mo, and W support stability, stronger bonding, and minimal distortion. The rigidity modulus and solid entropy of dopants, within the doping site, are key factors controlling stability. Moreover, low-concentration Ce doping experimentally confirmed the predicted distortions [69].

The previously mentioned atomically dispersed Co catalysts incorporated into Magnéli phase Ti_4_O_7_ ceramic membranes achieved uniform Co dispersion (Co^2+^/Co^3+^) while retaining high conductivity and stability. DFT calculations confirmed the role of Co active sites and strong metal–support interactions in generating ·OH and ^1^O_2_ radicals. This approach demonstrated energy consumption orders of magnitude lower than typical electrochemical treatments [58].

Sui et al. [55] conducted density functional theory (DFT) calculations to acquire theoretical insights into the impact of doping on the calculated adsorption energies of PFOS and the C–S bond length in PFOS on both modified and unmodified Ti_4_O_7_ anodes. Researchers used the Vienna Ab initio Simulation Package (VASP) to perform the calculations with pristine and Ti_4_O_7_ with 18 distinct elements incorporated (Ag, Al, Au, Bi, Co, Cr, Cu, Fe, Mn, Mo, Ni, W, Pt, Ce, Y, Ta, Zr, and Nb) to evaluate the dopants’ effect on MPs’ catalytic activity. An MP with five dopants (Ce, Y, Ta, Zr, and Nb) exhibits enhanced performance relative to undoped anodes (reduced adsorption energies and an increase in C–S bond length in PFOS). DFT studies have also revealed the implications of doping on the band structure of Ti_4_O_7_. For example, Wei et al. [51] investigated Ce-doping in Ti_4_O_7_ and predicted that the introduction of Ce^3+^ ions modified the electronic structure and increased the electrical conductivity, showing how first-principles calculations can predict the functional capabilities of doped Ti_4_O_7_ in practical applications. These calculations predict changes in the band gap and electronic interactions, critical in optimizing Ti_4_O_7_ for use as an electrocatalyst.

The importance of theoretical insights is outlined in the context of determining the optimal doping levels and types. The DFT approach allows for the prediction of how variations in dopant concentration can influence the robustness and efficiency of Ti_4_O_7_ in different environments, guiding experimental synthesis and optimization routes. The missing part in the current literature is the application of theoretical calculations (DFT and molecular dynamics), together with machine learning algorithms, on interactions between doped MPs and different pollutants. This type of calculation is crucial for understanding adsorption and/or degradation processes.

## 5. Perspectives and Outlook

The versatility of doped MP titanium suboxides places them at the forefront of future electrode materials in electrochemical applications. So far, their reported effectiveness in the degradation of emerging and persistent pollutants, such as PFAS and pharmaceuticals, warrants further scrutiny. Dopant-supported modulation of stability, conductivity, electronic structure, oxygen evolution potential, and reactive radical generation extends their use from environmental to energy-related applications.

Several research directions deem attention: (i) broadening the scope of contaminants to pesticides, dyes, and other persistent pollutants that would place TSOs as electrodes of choice for water treatment; (ii) conductivity modulation geared towards electrocatalytic processes, and energy conversion and storage, including the oxygen reduction reaction and 2e^−^ pathways for hydrogen peroxide production; (iii) modification of electronic structure for photocatalytic reactions; (iv) preparation of composites with solid supports for targeted applications; and (v) rational dopant selection, supported by density functional theory (DFT), can help establish favorable adsorption energies and reaction kinetics for both environmental and energy applications.

The prospects for future doped TSO applications rely on advances in material science to prevent dopant leaching. Under electrochemical operating conditions, partial leaching of dopants may occur due to redox cycling and local reorganization near the surface. This may alter the Ti^3+^/Ti^4+^ equilibrium and lead to lower conductivity and activity. The incorporation of dopants within composite frameworks overcomes these drawbacks by forming stronger bonds and thereby stabilizing the structure. Other strategies for surface modification aim towards thin coatings or the formation of composite materials with carbons or conductive polymers that stabilize dopant sites without compromising electronic structure. Prevention of leaching, coupled with controlled doping, provides the performance and stability necessary for targeted applications.

Furthermore, future studies should enable efficient substitution without loss in stability and porosity and simplify synthesis methods. The combination of computational and characterization methods is essential to model bond cleavage, pollutant oxidation, and electrokinetic intermediates.

Whenever there is a rush over novel and underinvestigated materials, researchers tend to unnecessarily overcomplicate synthesis and applications. Here, we might have a class of materials to actually suit several processes, from environmental and energy-related ones, justifying the rising number of studies, both theoretical and experimental. Look at the periodic table; a lot of research is still ahead.

## 6. Conclusions

Doping TSO has proven to be a viable strategy for producing materials with preserved intrinsic high conductivity and stability. The level of foreign atom incorporation is limited to <10% at. with higher doping levels, inducing irreversible changes to the original lattice and phase separation and resulting in lower activity. High-temperature reduction with H_2_, carbon, or Ti is the dominant route for producing pristine and doped TSOs with desirable conductivity and stability. Complete electrochemical oxidation of a myriad of pollutants is viable, including PFAS/PFOS, antibiotics, microbes, pharmaceutics, wastewater, etc. As this niche is developing, around 15 doped TSOs have been made experimentally so far, with DFT predicting sufficient stability for 11 more to be prepared.

## Figures and Tables

**Table 1:** Summary of doped MP and their applications or alterations in structural/thermal features.

Dopant		Application/Characteristics	Performance Metric	Author/ Reference
Ion/Atom	Conc.			
**Pt**	290 ppm	Photocatalytic hydrogen evolution reaction	50–100 times higher H_2_ evolution than plain anatase loaded with a similar amount of Pt	Wierzbicka et al., 2019. [53]
**Ce** **^3+^**	1–3 at%	Electrooxidation of perfluorooctane sulfonate (PFOS)	Oxidation rate 2.4× greater than that of the pristine Ti_4_O_7_ electrode	Lin et al., 2021. [54]
**Ce** **^3+^ Nb** **^5+^**	2.6 at% Ce 5.5 at% Nb	Electrooxidation of PFOS	Nb-TSO anode 1.8 times lower and Ce-TSO anode higher PFOS degradation rate than that of the Ti_4_O_7_ anode	Sui et al. 2025. [55]
**V**	nominally 1, 2, and 5 at%	Electrochemical oxidation of industrial wastewater	Decolorization >99 and chemical oxygen demand removal >98% of 100 mg L^−1^ of methyl orange	English et al., 2023. [56]
**Sm** **^3+^**	0.25, 0.5, 1%	Electrochemical removal of sulfamethazine	91.2% removal efficiency for sulfamethazine (0.25% Sm-Ti_4_O_7_ anode)	Tao et al., 2025. [57]
**Co** **^2+^ Co** **^3+^**	3.05 wt%	Sulfamethoxazole (SMX) removal	100% SMX (10 mg/L) removal with a high rate constant (34.07 min^−1^)	Jing et al. [58]
**Sn** **^4+^/Sn** **^2+^**	0.88%	Electrochemical oxidation of tetracycline	92% removal rate within 120 min (compared to 71.4% for pristine Ti_4_O_7_) and complete removal within 180 min	Jia et al., 2025. [59]
**Ce** **^3+^**	10 at% Ce	Monocycline (MNC) degradation within wastewater	100% MNC removal within 20 min; removal rate reduces from 100 to 98.5% after five cycles	Chen et al., 2024. [60]
**Sn** **^4+^**	0.5–5 at%, (best at 1 at%)	ORR for producing H_2_O_2_	H_2_O_2_ selectivity of 95.7%	Sun et al., 2024. [52]
**La**	1.60%	Electrooxidation of florfenicol (FLO)	Removal efficiency of FLO (>93.5%) within 20 degradation cycles	Xu et al., 2022. [61]
**Ru** **^2+^**	0.13 wt%	Electrochemical chlorine evolution reaction (ClER)	Active ClER both in 5 M NaCl (pH 2.3) and 0.1 M NaCl (pH 6.5) electrolytes. (Ru1-Ti_4_O_7_ catalysts outperform existing DSA materials—ClER TOF 17-fold and mass activity 23-fold greater)	Lee et al., 2024. [62]
**Nd**	1 wt%	Electrochemical disinfection and degradation of antibiotic resistance genes	Complete inactivation (>8.0-log inactivation) of antibiotic-resistant *Escherichia coli*	Zhang et al., 2024. [63]
**Pd, Cu**	Pd:Cu = 2:1 2 wt% Pd	Electrochemical oxidation and reduction of sulfamethoxazole (SMX)	Electrochemical reduction using the Pd-Cu/Ti_4_O_7_ achieved up to 96.1 ± 3.9% removal of SMX at a potential of −1.14 V/SHE and a permeate flux of 300 L m^−2^ h^−1^	Misal et al., 2020. [64]
**Al** **^3+^ and Ta** **^5+^**	Ti_4_O_7_ with 1 mol% Al_2_O_3_, Ti_4_O_7_ with 1 mol% Ta_2_O_5_ and Ti_4_O_7_ co-doped with 1 mol% Al_2_O_3_ and 1 mol% Ta_2_O_5_	Effect of doping on mechanical and electrical properties—mixed oxides	Co-doping simultaneously reduced the carrier concentration and enhanced carrier mobility (higher conductivity), but mechanical properties were not significantly improved	Geng et al., 2025. [65]
V, Cr, Fe	2 at%	Modification of the oxidation stability of MP in air	V-, Fe-Ti_4_O_7_ anodes—improved oxidation stability; Cr-Ti_4_O_7_—reduced lifetime	English et al., 2021. [41]
**mixed V** **^3+^/V** **^4+^/V** **^5+^**	6, 10, 18, 22 at%	Effects on structure and electrical conductivity	The synergistic interaction between carrier mobility and concentration leads to V-enhanced Ti4O7 conductivity.	Yuan et al., 2024. [66]
**V**	added amounts of V were 23, 27, 31, and 33 at%	Hollow V-doped MP, improved electrical conductivity and thermal stability	Improved the electrical conductivity 0.67 times, and the thermal stability (by increasing the reaction energy required to oxidize Ti_4_O_7_ to TiO_2_) of Ti_4_O_7_	Yuan et al., 2023. [67]

## Data Availability

Dataset available on request from the authors.
